# Tanshinone IIA attenuates renal injury during hypothermic preservation via the MEK/ERK1/2/GSK-3β pathway

**DOI:** 10.1186/s12906-021-03427-7

**Published:** 2021-10-08

**Authors:** Linhao Xu, Yizhou Xu, Zhoujing Zhu, Huiquan Gu, Chaofeng Chen, Jian Chen

**Affiliations:** 1 Department of Cardiology, Affiliated Hangzhou First People’s Hospital, Zhejiang University School of Medicine, Hangzhou, 310006 China; 2 Translational Medicine Research Center, Affiliated Hangzhou First People’s Hospital, Zhejiang University School of Medicine, Hangzhou, 310006 China; 3 School of Basic Medical Sciences and Forensic Medicine, Hangzhou medical college, No. 481 Binwen Road, Binjiang District, Hangzhou, 310053 Zhejiang China; 4 Zhejiang Provincial Center for Disease Control and Prevention, Hangzhou, 310051 China

**Keywords:** Tanshinone IIA, Hypothermic preservation, Celsior solution, Reactive oxygen species, Apoptosis

## Abstract

**Background:**

Oxidative stress-induced injury during hypothermic preservation is a universal problem that delays graft function and decrease the success of organ transplantation. Tanshinone IIA (Tan IIA) was reported to exhibit a variety of biochemical activities, including protection against oxidative stress. Therefore, the specific molecular pathway by which Tan IIA protects renal tissues during preservation was investigated in this study.

**Methods:**

In vivo study, Sprague-Dawley (SD) rats were divided into twelve groups and the kidneys were isolated and preserved in different solutions for 0, 24 or 48 h, respectively: control group (Celsior solution) and Tan II groups (Celsior solution containing 10, 50,100 μM). In vitro study, primary renal cell from SD rats was cultured which was treated H_2_O_2_ (800 μM) for 6 h to mimic oxidative stress injury. Four groups were finally divided: control group; H_2_O_2_ group; H_2_O_2_ + Tan IIA group; H_2_O_2_ + Tan IIA + G15 group.

**Results:**

In present study, we demonstrate data indicating that a significant increase in the superoxide dismutase (SOD) activity and a decrease in the reactive oxygen species (ROS) content were observed in the kidneys and renal cells preserved with Tan IIA compared with those preserved with the Celsior solution alone after 24 h and 48 h of hypothermic preservation (*P* < 0.01). The expression of phosphorylated mitogen-activated protein kinase kinase (MEK), phosphorylated extracellular signal-regulated protein kinases 1/2 (ERK1/2), phosphorylated glycogen synthase kinase-3β (GSK-3β) and cleaved caspase-3 was lower in the kidneys and renal cells preserved with Tan IIA than in those preserved with the Celsior solution alone after 24 h and 48 h of hypothermic preservation (*P* < 0.01). The mitochondrial morphology was rescued and adenosine triphophate (ATP) production and mitochondrial membrane potential were increased in the Tan IIA groups. Finally, Tan IIA also decreased cell apoptosis.

**Conclusion:**

It suggests that the supplementation of the standard Celsior solution with Tan IIA may significantly improve long-term kidney preservation. Tan IIA attenuated oxidative stress injury and decreased apoptosis levels via activation of the MEK/ERK1/2/GSK-3β signaling pathway during kidney hypothermic preservation.

**Supplementary Information:**

The online version contains supplementary material available at 10.1186/s12906-021-03427-7.

## Background

Renal transplantation is widely accepted as an effective treatment for renal disease [1]. The effective preservation of organs is a prerequisite for the success of organ transplantation. Currently, the most commonly used method of preservation for transplantation is static cold storage; however, some problems, such as oxidative stress injury and inflammation, still occur during organ preservation [2]. Therefore, the development of better organ preservation solution is a major goal that has attracted scientific attention.

Celsior solution is a kind of simulated extracellular solution that is widely used in the clinic for the cold preservation of different organs [3, 4]. However, a large degree of apoptosis is observed as the preservation time increases, and this increased apoptosis has a negative impact on the success rate of organ transplantation in postoperative patients. Although the mechanism of apoptosis during organ preservation is still unclear, it is generally believed that reactive oxygen species (ROS)-induced oxidative stress injury plays an important role [5]. However, under the condition of prolonged hypothermic preservation, the production of ROS increases due to mitochondrial dysfunction [6]. Therefore, the effect of standard preservation solutions has been improved by supplementation with ROS scavengers [7, 8].

Tanshinone IIA (Tan IIA) is extracted from the dry root of *Salvia miltiorrhiza*, and Tan IIA is the main active component of *Salvia miltiorrhiza*. Tan IIA exhibits multiple pharmacological activities, such as antioxidant, anti-inflammatory and anti-apoptosis [9–11]. In our previous study, Tan IIA was found to reduce oxidative stress injury during renal cryopreservation, and this effect might be related to the increase in superoxide dismutase (SOD) activity [7]. Furthermore, inhibition of the mitochondrial respiratory chain could lead to increased ROS production and reduced SOD enzyme activity [12]. Therefore, Tan IIA has an effect on increasing the activity of SOD, which may be related to the recovery of mitochondrial respiratory chain function; however, the underlying mechanism has not been reported.

In a previous study, Tan IIA reduced mitochondrial damage by increasing the phosphorylation of the glycogen synthase kinase-3 β (GSK-3β) protein by binding to the G protein-coupled estrogen receptor (GPER) [13, 14]. GSK-3β is a kinase that plays a critical role in regulating ROS production in mitochondria [15] and is phosphorylated via the mitogen-activated protein kinase kinase (MEK)/(extracellular signal-regulated protein kinases 1/2 (ERK1/2) signaling pathway [16]. Therefore, we hypothesized that Tan IIA could alleviate the renal injury induced by hypothermic preservation by activating the MEK/ERK1/2/GSK-3β signaling pathway.

## Methods

### Animals

Seventy-two male Sprague Dawley (SD) rats (200-220 g) were purchased from the Experimental Animal Center of Zhejiang University. These rats were housed underspecific pathogen-free condition at a temperature of 21 °C to 24 °C, humidity of 50 to 60% and a 12/12-h light/dark cycle. All the procedures were performed in experimental animal center of Zhejiang University and conducted with the approval of the local animal care committee (Zhejiang University). The study was carried out in compliance with the ARRIVE guidelines.

### Reagents

Pentobarbital sodium salt and dichloro-fluorescein (DCF) diacetate were purchased from Sigma-Aldrich (St. Louis, MO, USA). Tanshinone IIA (Tan IIA) was obtained from Zhejiang Institute for Drug Control (Hangzhou, Zhejing, China). G1 and G15 were purchased from ApexBio (Houston, TX, USA). The SOD assay kit was purchased from NanJin Jin Cheng Bioengineering Institute (NanJing, Jingshu, China). Annexin V−/PI staining kit was purchased from Yeasen Biotech (Pudong, Shanghai, China). The phospho-MEK^Ser221^, phospho-ERK1/2^Thr202^ and phospho-GSK-3β^Ser9^ antibodies were purchased from OmnimAbs (Alhambra, CA, USA), and the cleaved caspase-3 antibody was purchased from Cell Signaling Technology (Danvers, MA, USA). The Tamm-Horsfall protein, aquaporin-1 and nephrin were purchased from Affinity Biosciences (Cincinnati, OH, USA).

### Kidney hypothermic preservation

All the rats divided into eight groups using a randomized complete block design (*n* = 6 each) were anaesthetized by an intraperitoneal injection of 1% pentobarbital sodium salt (30 mg/kg, Sigma-Aldrich, Darmstadt, Germany). The kidneys of each rat were fully exposed, and the renal vessels were ligated to block the blood supply to the kidneys. The renal artery was cannulated using a Tibbs arterial cannula connected to a 50-mL syringe and was perfused with a 4 °C Celsior solution (mM: 100 NaOH, 15 KCl, 13 MgCl_2_, 0.25 CaCl_2_, 60 mannitol, 80 lactobionate, 30 histidine, 20 glutamate; pH = 7.4) or with a 4 °C Celsior solution containing 10, 50, or 100 μM Tan IIA. The kidneys were perfused until the solution effusing from the renal vein appeared clear. The kidneys were then removed and stored in different preservation solutions for 0, 24 or 48 h at 4 °C; the preservation solutions included Celsior solution or Celsior solution containing 10, 50, or 100 μM Tan IIA. For each animal, three different investigators were involved as follows: rats were divided by a first investigator (YZX) based on the randomization Table. A second investigator (JC) was responsible for the anaesthetic procedure, whereas a third investigator (LHX) performed the surgical procedure. Finally, the rats were sacrificed by decapitation. The primary endpoint of this study was defined as superoxide dismutase (SOD) activity and ROS concentration, Secondary endpoints were the expression of phospho-MEK^Ser221^, phospho-ERK1/2^Thr202^ and phospho-GSK-3β^Ser9^ detected by immunohistochemistry and western blot. The ultrastructural changes in mitochondria was observed by transmission electron microscopy**.** The sample size calculation was based on our previous study [7, 8], a total of six rats per group were considered necessary.

### Primary renal cell cultures

Kidneys were removed under sterile conditions from 1- to 2-day-old newborn rats anesthetized by the inhalation of isoflurane 1.3 to 1.5% isoflurane (Abbott Laboratories, North Chicago, USA). After anesthesia, the newborn rats were decapitated and the kidneys were dissected, and the renal capsule and pedicle were removed. Then, the kidneys were cut into small pyramids and incubated with phosphate-buffered saline containing 150 U/ml collagenase for 1 h at room temperature. The suspension was resuspended several times, filtered using a mesh size of 80 μm (180 mesh) and centrifuged at 1500 rpm for 5 min. The cell pellet was resuspended in Dulbecco’s modified Eagle’s medium (DMEM) supplemented with ITS liquid media supplement, 5% fetal bovine serum (Sigma), penicillin (100 U/mL), and streptomycin (100 lg/mL). The cell types were identified by the Tamm-Horsfall protein (for distal tubular cells), aquaporin-1 (for proximal tubular cells) and nephrin antibodies (for podocytes). The cells were used in all the experiments at approximately 60 to 70% confluence.

### Treatment of cells in culture

Different doses of Tanshinone IIA (125, 250, 500, or 1000 nM) and G15 (10, 50, 100, 200, or 500 nM) were added to the culture medium to assess the effects of these agents on cell viability (supplementary Fig. 2). The cells were treated with 0.8 mM H_2_O_2_ for 6 h to mimic oxidative stress injury. The cells were ultimately divided into four groups: the control group, H_2_O_2_ (800 μM) group, H_2_O_2_+ Tan IIA (250 nM) group, and H_2_O_2_ (800 μM) + Tan IIA (250 nM) + G15 (0.5 μM) group.

### MTT assay

Renal cell viability was assessed using the MTT assay. MTT (5 mg/ml) was added to the cells. After 4 h of incubation, the medium was discarded. DMSO (150 μL) was added to dissolve the resulting formazan crystals. The optical density was determined with a spectrophotometer (570 nm), and the data were normalized to solvent-treated cultures.

### Superoxide dismutase (SOD) activity and ROS concentration measurement

SOD activity was assessed by a SOD assay kit according to the manufacturer’s instructions as described our previous study [8]. The SOD assay kit (NanJin JinCheng ShenWu YanJiuSuo, NanJin, China) used the xanthine oxidase method. The kidneys were weighed, minced with scissors and homogenized into 10% tissue homogenate (homogenized for 3 × 10 s intervals on ice). From 5 to 10 mL of 10% tissue homogenate was centrifuged at 1000 r.p.m. for 10 min (4 °C) and the supernatant was transferred to a new tube; some supernatant was diluted to a concentration of 0.1 g/mL. Then, 20 μL of supernatant was added to the reaction system; this was incubated at 37 °C for 40 min, 2 mL of chromogenic agent was added and the mixture was incubated to room temperature for 10 min. The relative absorbance of the supernatants was immediately measured at 550 nm using a UV spectrometer. 20 μL of ddH_2_O was used instead of supernatants as a negative control. A 1 mg protein of a sample solution that established 50% inhibition was used to determine the SOD unit in an assay solution as 1 U.

To quantify the ROS level, renal tissue (five kidneys from each group) was homogenized in 0.01 M PBS. The tissue homogenates or renal cells were then centrifuged at 6000 rpm for 15 min (4 °C), and 5 μl of the supernatant was mixed with 55 μl HEPES (0.02 M) and 90 μl fresh DCF-DA (20 μM, Sigma-Aldrich, St. Louis, MO, USA) in a black, flat-bottomed 96-well plate and incubated at 37 °C for 30 min. The fluorescence intensity was determined with a plate reader (SynergyMx, BioTek, Vermont, USA) at an excitation wavelength of 485 nm.

### Annexin and Propidium iodide staining

After 6 h of H_2_O_2_ treatment, the cells were harvested, and the apoptosis levels were detected using an Annexin V−/PI staining kit (Yeasen Biotech Co., Ltd). First, the cells were collected and washed with PBS. Second, the cells were resuspended at 2 × 105 cells/mL in 100 μL of binding buffer and then incubated with 5 μL of Annexin V-FITC and 10 μL of PI staining solution in the dark for 20 min at room temperature. Third, 400 μL of binding buffer was added. The samples were analyzed using a Beckman CytoFLEX with CytExpert software and assessed according to the percentage of Annexin V-PI-positive cells.

### Immunohistochemistry and terminal transferase-mediated dUTP nick end labeling (TUNEL) staining

Three kidneys from each group were fixed in 4% paraformaldehyde solution for 24 h and embedded in paraffin. For immunohistochemistry, serial sections were deparaffinized, re-hydrated and immersed in 10 M citric acid (pH 6.0) to prevent epitope masking due to fixation. The sections were then stained with primary antibodies against phospho-MEK^Ser221^, phospho-ERK1/2^Thr202^ and phosphor GSK-3β ^Ser9^ by incubation with the antibodies overnight at 4 °C. The tissue sections were then incubated horseradish peroxidase labeled goat anti-rabbit IgG (Abacm, MA, USA). Immunolabeling was visualized with 0.05% diaminobenzidine (DAB) plus 0.3% H_2_O_2_ in PBS. The sections were subsequently counterstained with hematoxylin before being dehydrated with ethanol and xylene and coverslipped with Permount. The staining scores for renal tissue were determined under high (× 400) magnification with a panoramic view as previously described [17]. The intensity of the immunoreactivity was scored as 0, negative; 1+, weak; 2+, moderate; or 3+, strong. An H-score was calculated according to the following formula:

H-score = (percentage of cells with weak staining× 1) + (percentage of cells with moderate staining× 2) + (percentage of cells with strong staining× 3).

A digital microscope (Zeiss microscope axiophot 2, Zeiss, Germany) was used to analyze 3 randomly selected fields from each section at 400× magnification to identify the positive cells. Apoptosis was detected by the in situ TUNEL technique, using an ApopTag1 kit (Millipore Corporation, Billerica, MA) and following the procedures recommended by the manufacturer.

### Western blot

Renal tissues and renal cells were homogenized with ice-cold radioimmunoprecipitation assay buffer, and the protein concentration was assessed by the BCA Protein Assay Reagent Kit (Thermo Fisher Scientific, Waltham, MA, USA). Thirty micrograms of protein was separated by SDS-PAGE and transferred to nitrocellulose filter membranes (Millipore Corporation) at 100 V for 1 h by the wet transfer method. The membranes were blocked with 5% nonfat dry milk at 37 °C for 1 h and incubated with the relevant primary antibodies at 4 °C overnight. Then, the membranes were incubated with horseradish peroxidase-conjugated secondary antibodies, and the immunoreactive bands were visualized using an ECL kit (Thermo Fisher Scientific, Waltham, MA, USA).

### Transmission electron microscopy

Transmission electron microscopy was used to investigate the ultrastructural changes in mitochondria. After 0 h, 24 h or 48 h of hypothermic preservation, the renal tissue was diced into 1 mm^3^ pieces and further fixed using 2.5% glutaric dialdehyde for 4 h. The pieces were postfixed in 2% osmium tetroxide for 30 min, dehydrated in graded alcohol, transferred to propylene oxide, and gradually embedded in blocks of Epon 812 resin for 2 days at 60 °C. Eighty-nanometer sections were collected using a diamond histoknife (Diatome) on an Ultracut E microtome (Leica) and then mounted on a copper mesh and stained with uranyl acetate and lead nitrate. The ultrastructural changes in mitochondria was identified by a transmission electron microscope (Hitachi H-7700).

### Isolation of mitochondria and measurement of mitochondrial function

Mitochondria were isolated from renal tissues using the discontinuous Percoll density gradient method [18]. Isolated mitochondria suspensions was immediately detected. The rate of ATP production was determined by a bioluminescence assay kit (Sigma-Aldrich; Stock No. FL-AA) as described [19]. The mitochondrial membrane potential was assessed using the lipophilic cationic carbocyanine probe JC-1 [20]. More detailed information is included in the Supplementary material.

### Data analysis

The data are expressed as the mean ± standard deviation. Significant differences were determined by one-way ANOVA, followed by Tukey’s test as the post hoc test for multiple group comparisons for the in vitro experiments and by two-way ANOVA factoring time and treatment type for the animal experiments. A *p* value of < 0.05 was regarded as statistically significant.

## Results

### Tan IIA decreased the reactive oxygen species (ROS) level and increased the superoxide dismutase (SOD) activity

**Fig. 1:**
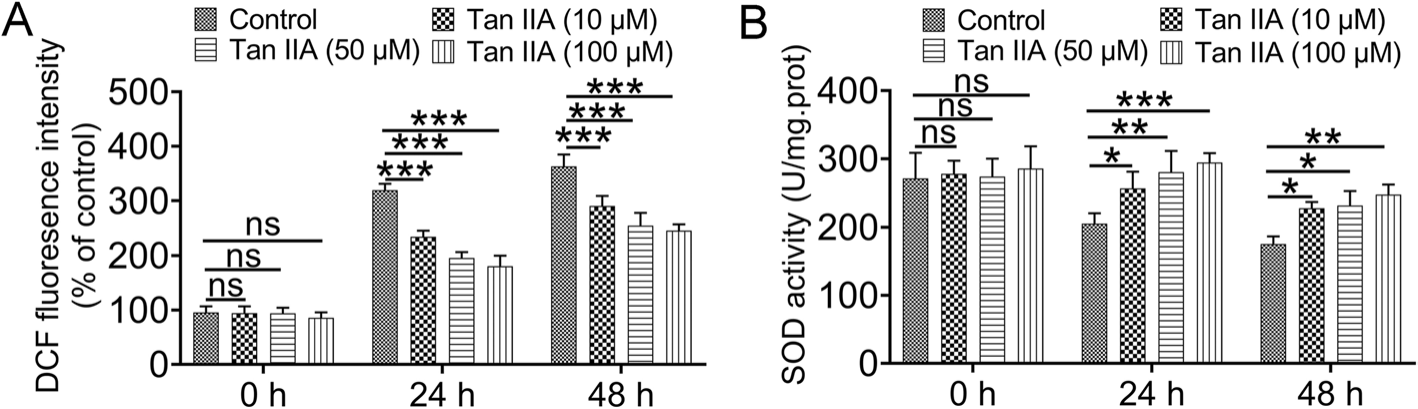
Reactive oxygen species (ROS) production was reduced and superoxide dismutase (SOD) activity was increased after Tan IIA treatment. (**A**) The intensity of DCF fluorescence, which indicated the production of ROS in each group; (**B**) the activity of SOD in each group. The values are the mean ± standard deviation of 5 rats per group. ns > 0.05; *P < 0.05; **P < 0.01; ***P < 0.001

Based on the results in Fig. 1, two-way ANOVA revealed that ROS production and SOD activity were both affected by the duration of hypothermic preservation and Tanshinone IIA treatment. Post hoc Tukey’s test indicated that the ROS levels and SOD activity in the renal tissues were not significantly difference between control and Tan IIA groups at 0 h, however, ROS levels was clearly elevated and SOD activity was obviously reduced after 24 h and 48 h of hypothermic preservation when compared with 0 h hypothermic preservation groups. Meanwhile, 24 or 48 h Tan IIA treatment had an effect on reducing the ROS content compared with the control at 24 or 48 h hypothermic preservation (*P* < 0.001). Furthermore, the SOD activity was also significantly increased by Tan IIA treatment with 24 h or 48 h compared with the control at 24 and 48 h hypothermic preservation, which was consistent with our previous study [7].

### Tan IIA rescue the ultrastructure and function of mitochondrial

**Fig. 2:**
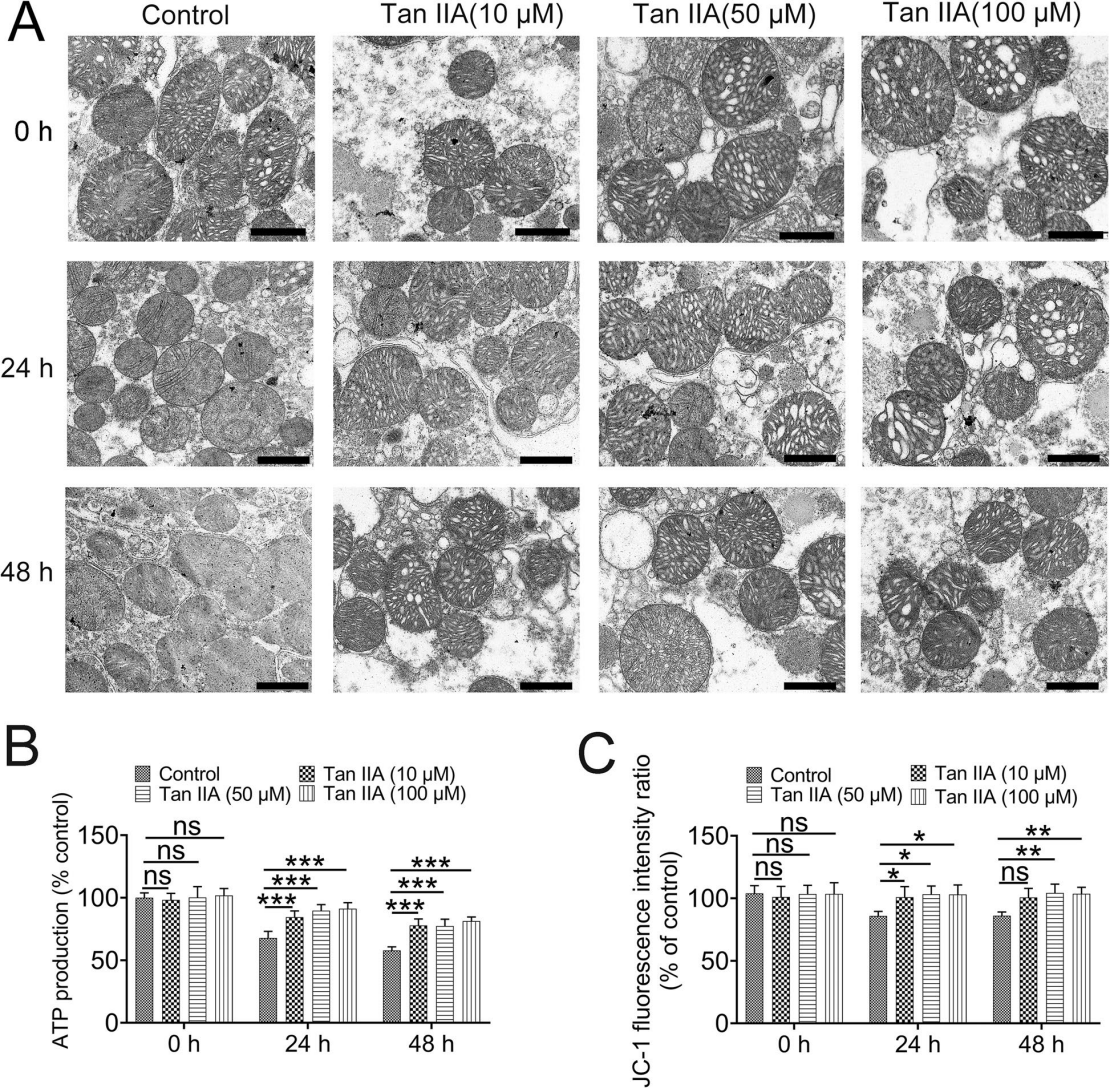
Impaired mitochondrial morphology and function were rescued by Tan IIA. (**A**) The morphology of mitochondria in the renal tissue. Control group and Tan IIA treatment groups at 0 h displayed an intact structure with clear cristae, however, fewer cristae were observed in the mitochondria in the control group after 24 h and 48 h of hypothermic preservation, and Tan IIA treatment preserved more intact mitochondria with discernable cristae. Scale bar: 1 μm. Mitochondrial ATP production (**B**) and mitochondrial membrane permeability (**C**) were partially reduced by Tan IIA. ns > 0.05; *P < 0.05; **P < 0.01; ***P < 0.001; *n* = 5

As discussed above, SOD plays an important role in mitochondrial function. Therefore, to investigate whether the function of mitochondria was affected by Tan IIA, the morphology and some functional parameters of mitochondria were analyzed. As shown in the electron micrographs in Fig. 2A, the mitochondria found in the control group and Tan IIA treatment groups with 0 h hypothermic preservation displayed an intact structure with clear cristae, however, most of the mitochondrial cristae disappeared in the control group after 24 h or 48 h hypothermic preservation. Meanwhile, the cristae were more intact and easier to observe in the 24 h or 48 h Tan IIA treatment group (Fig. 2A). Statistics analysis indicated that 24 h or 48 h hypothermic preservation could lead to a significant reduction in ATP production (Fig. 2B) and mitochondrial membrane permeability (Fig. 2C), and these effects were rescued by Tan IIA treatment.

### Tan IIA reduce renal cell apoptosis via suppressing caspase-3 activation

**Fig. 3:**
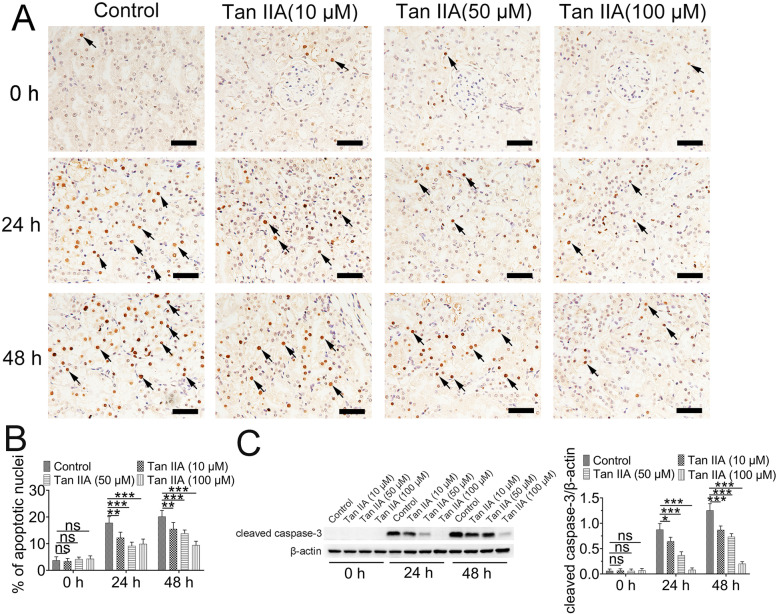
Tan IIA alleviated apoptosis. (**A**) Representative images of apoptotic cells in the renal tissue, as analyzed by the TUNEL assay, Scale bar: 50 μm. (**B**) Statistics analysis showed that fewer apoptotic cell in renal tissue of control group and Tan IIA groups at 0 h, however, 24 h and 48 h of hypothermic preservation triggered an increase in the number of apoptotic cells (arrow), whereas Tan IIA reduced the number of apoptotic cells, *P < 0.05; **P < 0.01; ***P < 0.001; *n* = 5. (**C**) The expression of cleaved caspase-3 was reduced by Tan IIA. ns > 0.05; *P < 0.05; **P < 0.01; ***P < 0.001; *n* = 3

TUNEL staining revealed fewer apoptotic cell in renal tissue of control group and Tan IIA groups at 0 h (Fig. 3A and B), meanwhile, the expression of cleaved caspase-3 did not easily observed in groups with 0 h hypothermic preservation (Fig. 3C). However, the number of apoptotic cell and cleaved caspase-3 levels were also obviously increased in control groups after 24 h or 48 h hypothermic preservation, which was significantly decreased by 24 h or 48 h Tan IIA treatment (Fig. 3C).

### Tan IIA activated the MEK/ERK1/2 pathway during renal hypothermic preservation

**Fig. 4:**
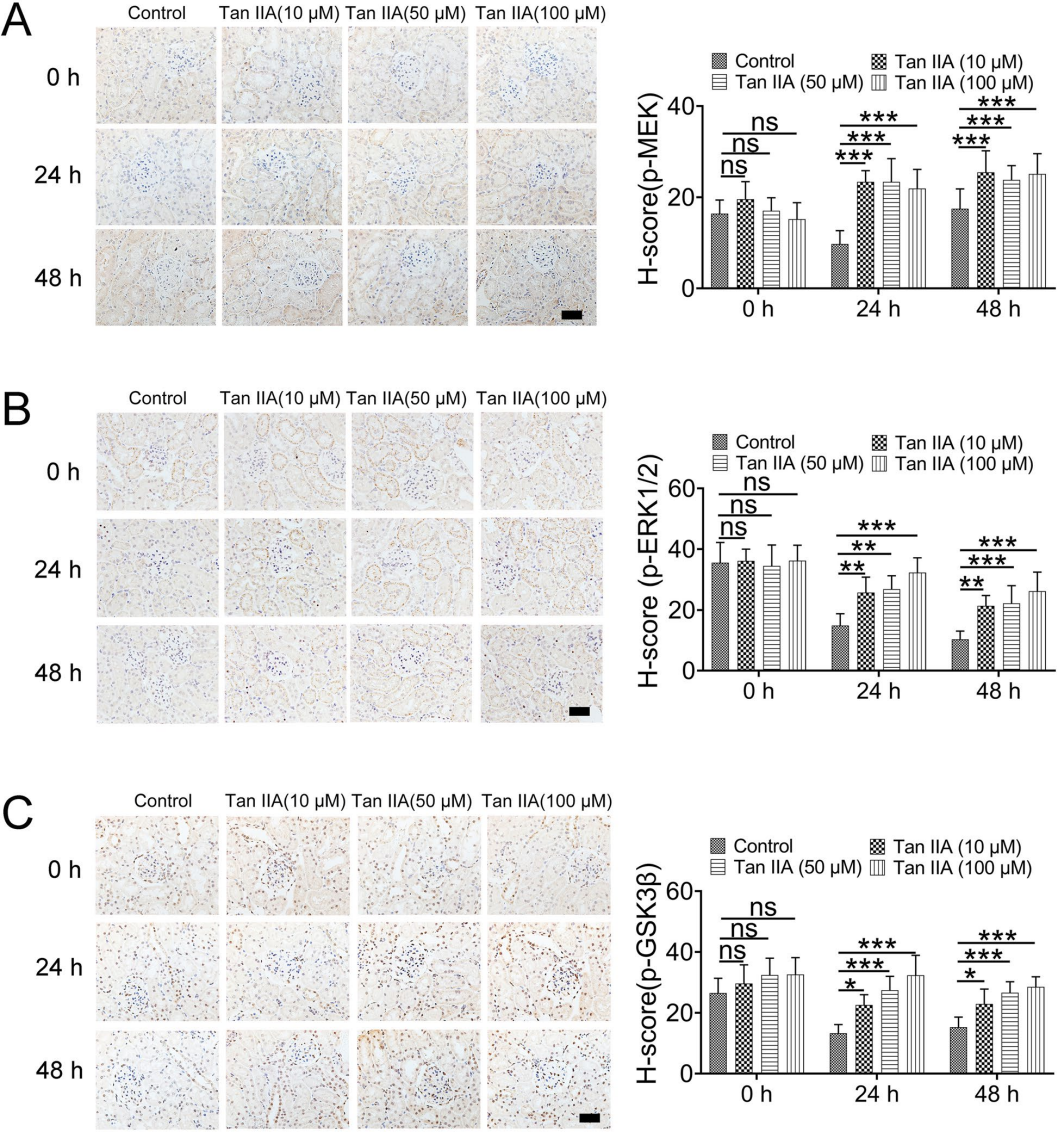
Tan IIA increased the expression of p-MEK (**A**), p-ERK1/2 (**B**) and p-GSK-3β (**C**), as assessed by immunohistochemical analysis. The data represent the mean ± standard deviation of nine sections per each group. Scale bar: 50 μm. ns > 0.05; *P < 0.05; **P < 0.01; ***P < 0.001

**Fig. 5:**
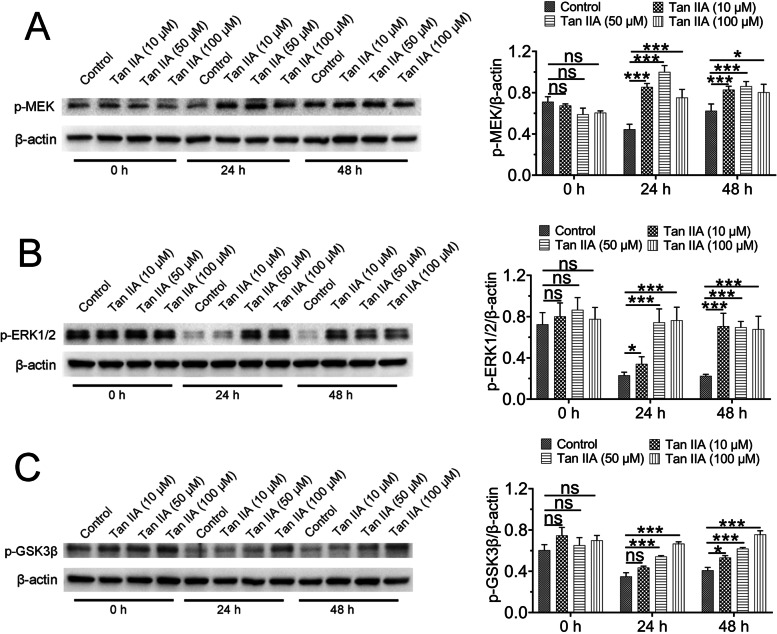
Tan IIA increases the expression of p-MEK (**A**), p-ERK1/2 (**B**) and p-GSK-3β (**C**), as detected by immunoblotting. The data represent the mean ± standard deviation of three rats per each group. ns > 0.05; *P < 0.05; **P < 0.01; ***P < 0.001

In this study, two methods, including immunohistochemistry and western blot assays, were used to assess the expression of p-MEK, p-ERK1/2 and p-GSK-3β. As shown in Figs. 4 and 5, the effects on the expression of p-MEK, p-ERK1/2 and p-GSK-3β did not altered difference between control and Tan IIA groups with 0 h hypothermic preservation, however, the expression of these three proteins was reduced in control group and Tan IIA treatment increased the phosphorylation of ERK1/2, MEK and GSK-3β after 24 h or 48 h hypothermic preservation.

### Cell viability was rescued and apoptosis was inhibited by tan IIA

To investigate whether Tan IIA activates the MEK/ERK1/2/GSK-3β signaling pathway by binding to GPER, the GPER inhibitor G15 was added to primary renal cells. To confirm the renal cell-specific phenotypes, immunofluorescence staining was performed on the cultured cells using several renal cell markers, including Tamm-Horsfall protein, aquaporin 1 and nephrin, which are expressed by different tubular and podocyte cell populations. As showed in supplementary Fig. 1, approximately 64.06 ± 7.39% of cells were positive for nephrin, which suggested that a large percentage of the cells were podocytes; furthermore, there was approximately 14.00 ± 1.34% distal tubular cells and 18.50 ± 3.70% proximal tubular cells. As shown in supplementary Fig. 2, treatment with a certain concentration of H_2_O_2_ (800 μM) for 6 h reduced cell viability, while G15 (0.5 μM) or Tan IIA (250 nM) alone did not affect cell viability.

**Fig. 6:**
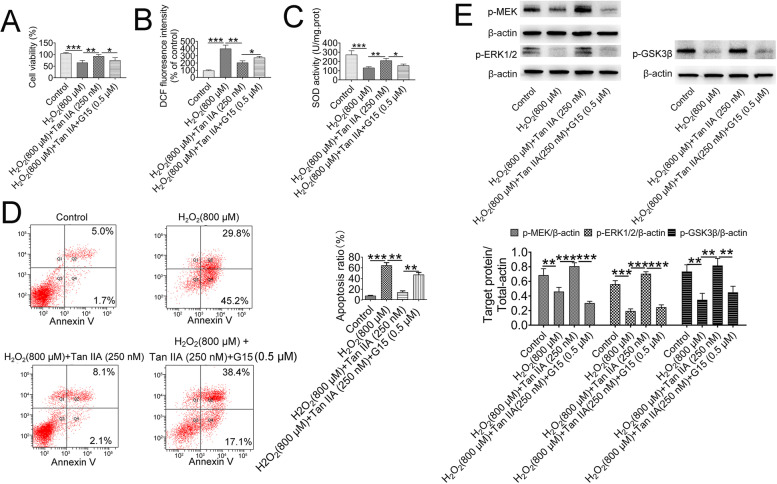
Tan IIA rescued cell viability and inhibited apoptosis via activating MEK/ERK1/2/GSK-3β pathway. (**A**) Cell viability was assessed by MTT assay; (**B**) reactive oxygen species (ROS) production; (**C**) superoxide dismutase (SOD) activity; (**D**) Annexin V-FITC/PI staining, as measured by flow cytometry, revealed a significant increase in the apoptotic cell ratio in the H_2_O_2_ treatment group, and this increased apoptosis was reduced by Tan IIA. (**E**) Tan IIA increased the expression of p-MEK, p-ERK1/2 and p-GSK-3β, as detected by immunoblotting. G15 could diminish the expression of these proteins induced by Tan IIA. The data represent the mean ± standard deviation of three independent experiments per each group. *P < 0.05; **P < 0.01; ***P < 0.001

However, Tan IIA reversed the H_2_O_2_-induced effects on cell viability (Fig. 6A). Similar to the in vivo study, Tan IIA also decreased the ROS level and increased the SOD activity, and these effects were abolished by G15 supplementation (Fig. 6B and C). Furthermore, the flow cytometry results showed that after H_2_O_2_ treatment, the percentage of apoptotic cells was approximately 64.63 ± 9.11%, which was dramatically increased compared with that of the control cells (7.96 ± 1.25%, Fig. 6D); in addition, the percentage of apoptotic cells was reduced by Tan IIA treatment (14.60 ± 4.50%, Fig. 6D). Similar to the MTT test, the reduction in the percentage of apoptotic cells observed in the Tan IIA group was increased again in the H_2_O_2_+ Tan IIA + G15 group (47.40 ± 7.23%, Fig. 6D). This result suggests that the protective effect of Tan IIA in suppressing apoptosis is associated with the binding of Tan IIA to GPER.

### Tan IIA activated the MEK/ERK1/2/GSK-3β pathway in primary renal cells

Similar to the in vivo study, the expression of p-MEK, p-ERK1/2 and p-GSK-3β was significantly decreased in H_2_O_2_ groups. Post hoc Tukey’s test revealed that the expression of these three proteins was increased after Tan IIA treatment and reduced after H_2_O_2_ + Tan IIA + G15 treatment in primary renal cells (Fig. 6E).

## Discussion

In recent years, it has been observed that prolonged cold storage and warm ischemia of donor kidneys increase the incidence of delayed graft function after transplantation [21]. Therefore, the method of cold static storage, as the most widely used preservation method in kidney transplantation, was needed to reduce injury during hypothermia ischemia. Although multiple harmful consequences occur, excessive ROS production plays an important role in cell damage during hypothermia [22]. In the present study, the ROS levels in renal tissue were indeed increased after 24 h and 48 h of hypothermic preservation when compared with groups at 0 h hypothermic preservation, and these increased levels were accompanied by a reduction in the SOD activity. Moreover, Tan IIA could increase the SOD activity and decrease ROS production, which was consistent with our previous studies [7, 23]; however, the underlying mechanism was not determined.

As discussed above, SOD is the major antioxidant enzyme for scavenging ROS by converting superoxide anions into hydrogen peroxide [24]. SOD activity is associated with mitochondria, which are also major sources of ROS. Therefore, we believe that the effect of Tan IIA on rescuing SOD activity and reducing ROS production was associated with mitochondria. According to our results, the morphology of mitochondria was destroyed, and the function of mitochondria was also impaired after hypothermic preservation; however, Tan IIA could indeed rescue mitochondrial function, including increasing ATP production and mitochondrial membrane permeability. It is well known that ATP synthesis is dependent on the mitochondrial respiratory chain [25]. Under physiological conditions, a low concentration of ROS is maintained by the action of these oxidoreductases; however, complex I/III activity in the electron transport chain is impaired during hypothermic preservation, and this could result in the enhanced production of ROS and the depletion of ATP [26]. Therefore, we hypothesized that the effect of Tan IIA in reducing ROS and ATP was associated with rescuing the mitochondrial respiratory chain.

It is well known that Ca^2+^ homeostasis plays an important role in regulating ATP synthesis and complex I activity [27], however, a massive influx of Ca^2+^ into the mitochondrial matrix could inhibit electron transport between complexes I and III [28]. Moreover, the accumulation of Ca^2+^ in mitochondria was observed during organ preservation [26]. In this process, the mitochondrial permeability transition pore (mPTP) was opened, which facilitated Ca^2+^ influx and dissipated the mitochondrial membrane potential, resulting in cell death [29]. In present study, ATP level and mitochondrial membrane potential was indeed decreased after 24 h or 48 h hypothermic preservation which suggested that Ca^2+^ was accumulated in mitochondria. Meanwhile, Tan IIA increased the ATP level and mitochondrial membrane potential and had beneficial effects on rescuing renal cell activity and decreasing cell apoptosis in the in vitro study. These results suggested that Tan IIA alleviates the mitochondrial injury which was associated with blocking mPTP channel and reducing of Ca^2+^ influx. Although the effect of Tan IIA on inhibiting mPTP channel opening had been identified according to previous work [30], the molecular mechanism is still elusive.

Glycogen synthase kinase-3 β (GSK-3β) is a kinase that is present in the cytoplasm and mitochondria of a wide variety of cells. In general, GSK-3β can bind to mPTP, resulting in an influx of Ca^2+^ and an increase in ROS production [16]; however, the phosphorylation of GSK-3β separates it from the mPTP subunit and closes the mPTP channel [31]. Previous work demonstrated that the expression of GSK-3β was reduced during hypothermic preservation; however, the phosphorylation of GSK-3β was not altered [32]. Therefore, increasing GSK-3β phosphorylation might be a potential strategy to attenuate ROS-induced injury during hypothermic preservation. According to a previous study, Tan IIA can reduce mitochondrial damage by increasing the phosphorylation of GSK-3β [13]; therefore, we hypothesized that Tan IIA could accelerate the phosphorylation of GSK-3β during hypothermic preservation and attenuate mitochondrial damage. In the present work, it was observed that Tan IIA can indeed reduce mitochondrial damage and cell death, and these effects are related to the phosphorylation of GSK-3β. However, the molecular mechanism by which Tan IIA induces GSK-3β phosphorylation is still unclear.

Pharmacological studies have shown that Tan IIA has multiple biological sites with which it can bind to the G protein-coupled estrogen receptor (GPER) on the cell membrane [14]. Many studies have shown that the GPER is involved in the regulation and control of a variety of biological activities, including glucose and lipid metabolism [33]. However, a recent study found that the activation of the GPER receptor can reduce ischemia-reperfusion injury, and this effect might be related to the phosphorylation of GSK-3β [16]. Therefore, we speculate that the Tan IIA-induced GSK-3β phosphorylation is related to the interaction between Tan IIA and GPER. When Tan IIA binds to GPER, it activates many downstream pathways, including the protein kinase (MEK)/extracellular signal-regulated protein kinases 1/2 (ERK1/2) signaling pathway [34]. In our study, the levels of phosphorylated GSK-3β, MEK and ERK1/2 were increased after Tan IIA treatment; however, G15, a GPER antagonist, eliminated the beneficial effect of Tan IIA in activating the MEK/ERK1/2/GSK-3β pathway and increased apoptosis. Tan IIA clearly reduced renal injury following hypothermic preservation.

## Conclusion

In conclusion, the present study demonstrated that the supplementation of the standard Celsior solution with Tan IIA may significantly improve the consequences of kidney preservation by attenuating oxidative stress and decreasing cell apoptosis. The underlying mechanism might be associated with the suppression of the MEK/ERK1/2/GSK-3β signaling pathway. Further efforts are required to explore the specific mechanism involved.

## Supplementary Information

**Additional file 1.**

## Data Availability

The datasets generated and analyzed during the current study are available from the corresponding author on reasonable request.

## Abbreviations

ATP: Adenosine triphophate
DAB: Diaminobenzidine
DCF: Dichloro-fluorescein
DMEM: Dulbecco’s modified Eagle’s medium
ERK1/2: Extracellular signal-regulated protein kinases 1/2
GPER: G protein-coupled estrogen receptor
GSK-3β: glycogen synthase kinase-3β
mPTP: Mitochondrial permeability transition pore
MEK: Mitogen-activated protein kinase kinase
ROS: Reactive oxygen species
SD: Sprague Dawley
SOD: Superoxide dismutase
Tan IIA: Tanshinone IIA
