# Supplementary material for: Tanshinone IIA attenuates renal injury during hypothermic preservation via the MEK/ERK1/2/GSK-3β pathway
Source: BMC Complement Med Ther. 2021 Oct 8;21:257. doi: 10.1186/s12906-021-03427-7 (PMC8501657; doi:10.1186/s12906-021-03427-7)
Supplement: Supplementary file 1 — Additional file 1. [file 12906_2021_3427_MOESM1_ESM.doc]

**Supplementary information**

**Supplementary Methods**

***Isolation of mitochondria from renal tissue***

Mitochondria were isolated from renal tissue using the discontinuous Percoll density gradient method (Sims and Anderson, 2008). Briefly, renal homogenate was made in ice-cold 12% Precoll solution and centrifuged at 30700 g, 4°C, for 5 minutes. The upper half of the liquid was aspirated to a new tube on ice, which included a layer of material consisting predominantly of myelin. The remainder of the centrifuged material in the centrifuge tube on ice was retained. Then, an equal volume of 14% Percoll solution was added into the material removed from the upper portion of the tube and centrifuged again at 30,700g at 4 °C for 5 min. The upper half of the centrifuged material was aspirated and discarded. The lower half with the comparable fraction retained from the ﬁrst centrifugation step was pooled. 20 μl of 50mg/ml digitonin solution was then added. Third, the digitonin-treated fraction (up to 3 ml per tube) was then layered with a glass Pasteur pipette on previously prepared discontinuous gradients consisting of 19% Percoll layered over 40% Percoll and was centrifuged at 30,700g at 4°C for 10 min. Fourth, using a glass pasteur pipette, successively removing the upper two layers of the gradient. The Percoll solution overlying the enriched mitochondrial fraction (band 3) was then removed into new tube and 4 volumes isolation buffer was added. Finally, after centrifuging at 16,700g at 4 °C for 10 min, the supernatant was aspirated and discarded. A small amount of material consisting of the mitochondria would be left at the base of the tube. After collection, the mitochondrial were immediately detected mitochondrial function.

***Mitochondrial Functional Assays***

The rate of ATP production was measured using a bioluminescence assay kit (Sigma–Aldrich, Stock No. FL-AA) as described (Hamada et al., 1998). Briefly, isolated mitochondria were immediately incubated for 5 min at room temperature with ATP reaction mixture. Luminescence signal was assessed on a plate reader (SynergyMx, BioTek, Vermont, USA). The ATP level was calculated as described in the technical bulletin of the kit and normalized by protein concentration.

***Fluorimetric analysis of mitochondrial membrane potential***

Changes in mitochondrial membrane potential were assessed by lipophilic cationic carbocyanine probe JC-1. Isolated mitochondria sample was incubated with JC-1 staining buffer according to the manufacturer’s instruction(Sigma, isolated mitochondria staining kit). The fluorescence intensity of JC-1 aggregates was detected with 520-nm emission filters, where the JC-1 monomer was measured with 485-nm excitation and 520-nm emission filters using a BMG Novo Star Galaxy spectroflurimeter. The fluorescence intensity ratio of aggregates to monomers was calculated as a indicator of mitochondrial membrane potential.

***Immunoﬂuorescence***

For immunoﬂuorescence, Cells in slides were ﬁxed in 4% paraformaldehyde for 4 h. Then, the slides of cultured cells were washed in 0.01 M PBS containing 0.3% Triton X-100 (pH 7.4, PBS-T), then immersed in 2% normal horse serum in PBS for 120 min at 37 °C, Slides were stained with primary antibodies against Tamm-Horsfall protein, aquaporin-1 and nephrin antibodies at 4 °C overnight. Then, Slides were rinsed in PBS and incubated with FITC (Rhodamine)-conju-gated goat anti-rabbit IgG secondary antibody (Invitrogen, CA, USA) for 1 h at 37 °C, and observed with a ﬂuorescence microscope (Leica).

**Supplementary Figures**

The cell types were identified by the Tamm-Horsfall protein (for distal tubular cells), aquaporin-1 (for proximal tubular cells) and nephrin antibodies (for podocytes). As showed in supplementary Figure 1, approximately 64.06 ± 7.39% of cells were positive for nephrin, which suggested that a large percentage of the cells were podocytes; furthermore, there was approximately 14.00 ± 1.34% distal tubular cells and 18.50 ± 3.70% proximal tubular cells.


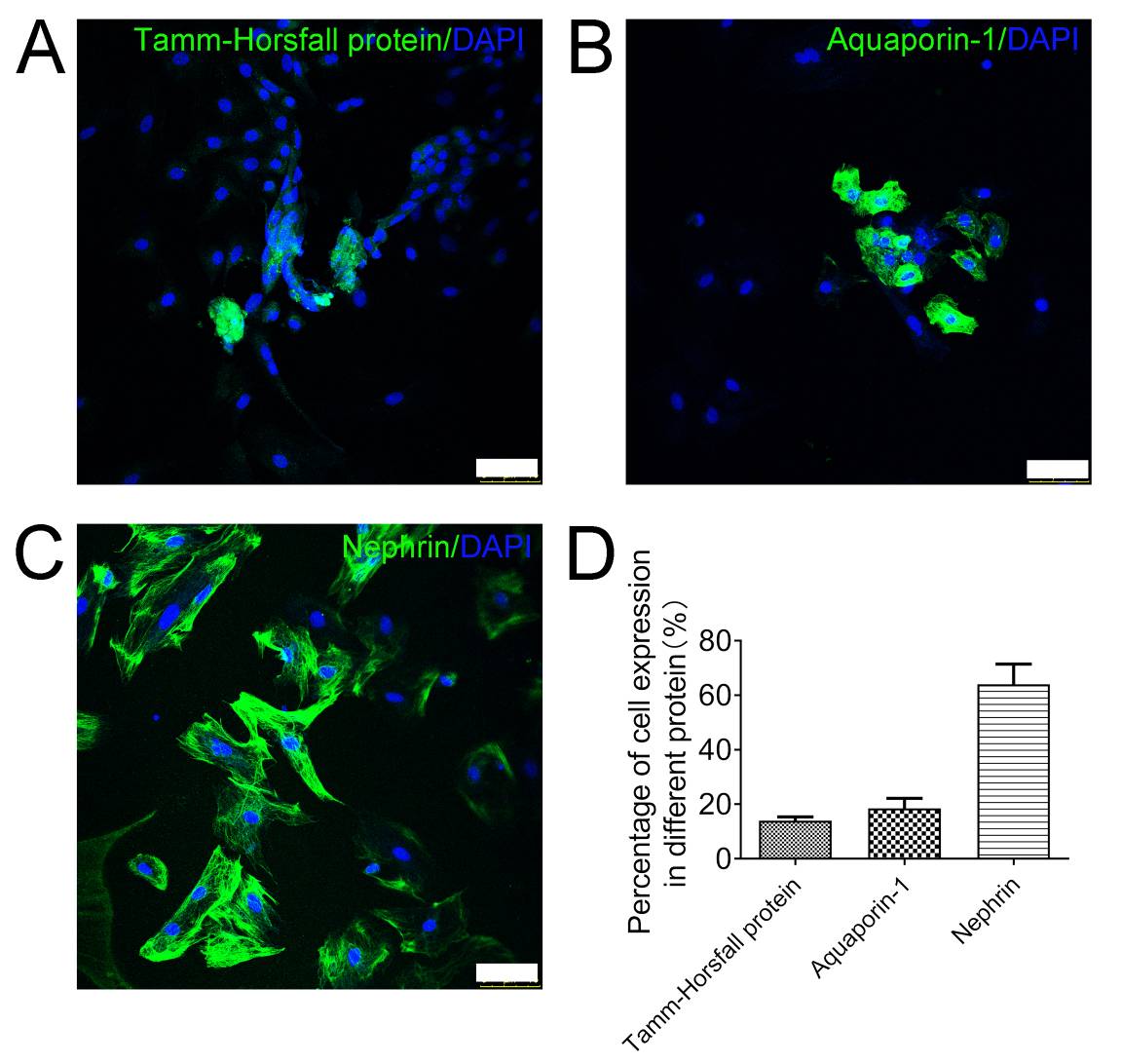
Supplementary Figure 1. Characterization of specific renal cells among the total renal cells isolated from rat. Cell-type specific antibodies (A: Tamm-Horsfall protein; B: Aquaporin-1;C: Nephrin) were used to identify distal tubular cells (A), proximal tubular cells (B) and podocytes (C) in different passages cultured renal cells from rats kidneys. (D) The quantification percentage of different cell type. n=3 scale bar: 75 μm.

Different doses of Tanshinone IIA (125, 250, 500, or 1000 nM) and G15 (10, 50, 100, 200, or 500 nM) were added to the culture medium to assess the effects of these agents on cell viability. As shown in supplementary Figure 2, treatment with a certain concentration of H_2_O_2_ (800 μM) for 6 h reduced cell viability, while G15 (0.5 μM) or Tan IIA (250 nM) alone did not affect cell viability.


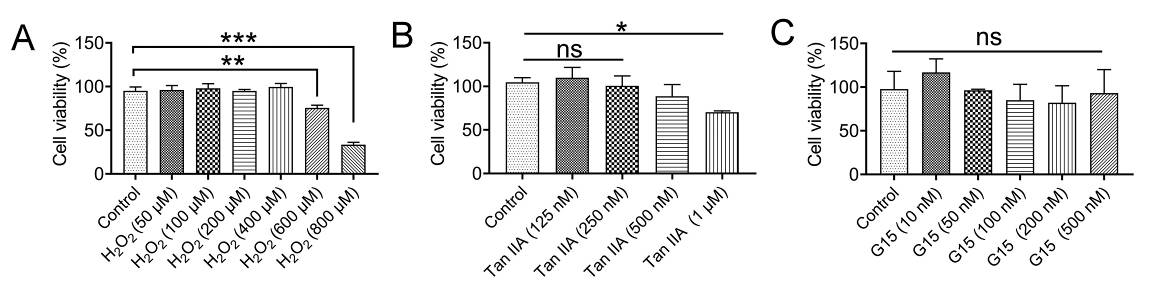


Supplementary Figure 2. Different dosage of H_2_O_2_ (A), Tan IIA (B) and G15 (C) affect cell viability.

Uncropped western blots of Fig 3C.

**
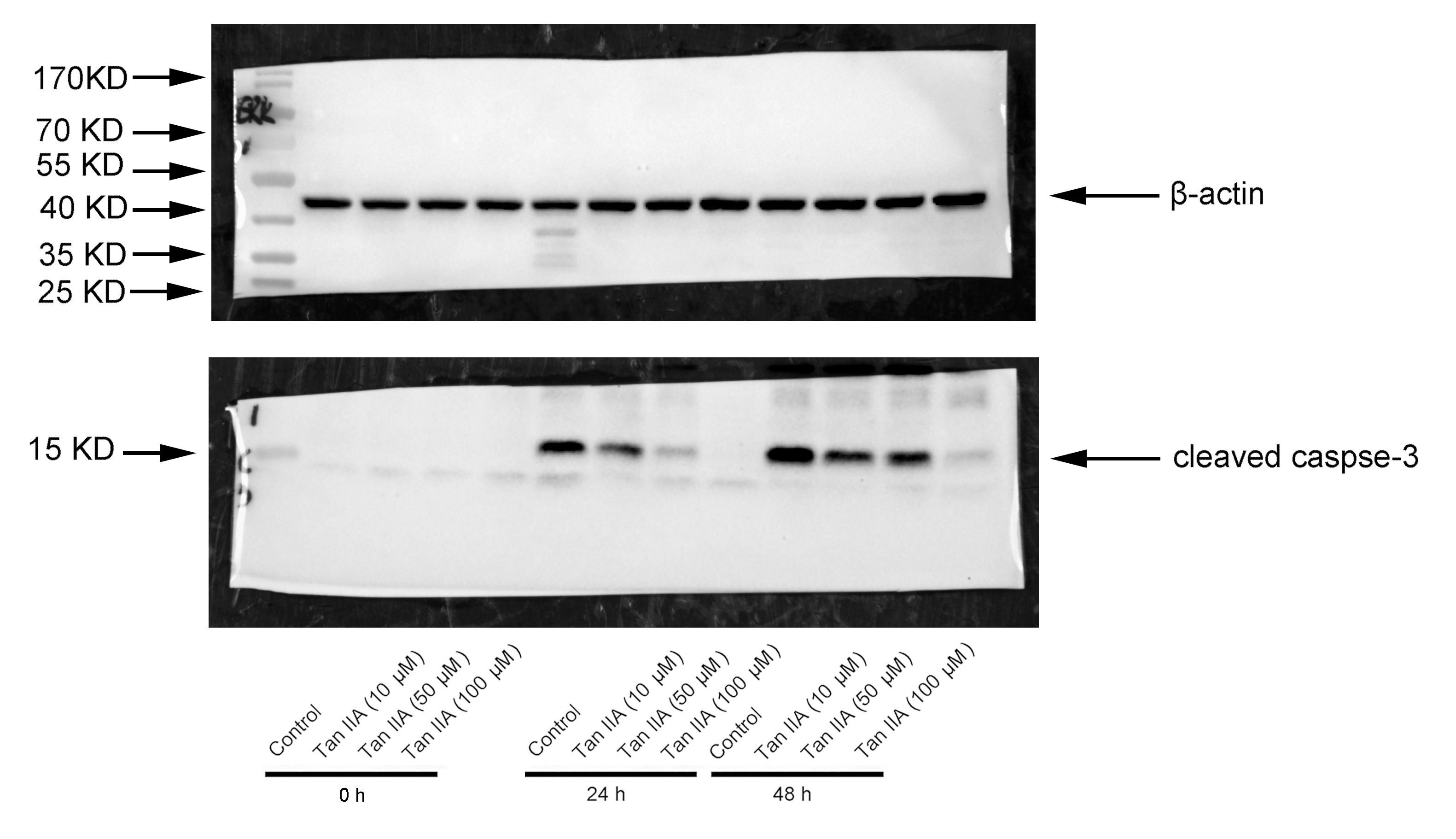
**

Uncropped western blots of Fig 5A.

**
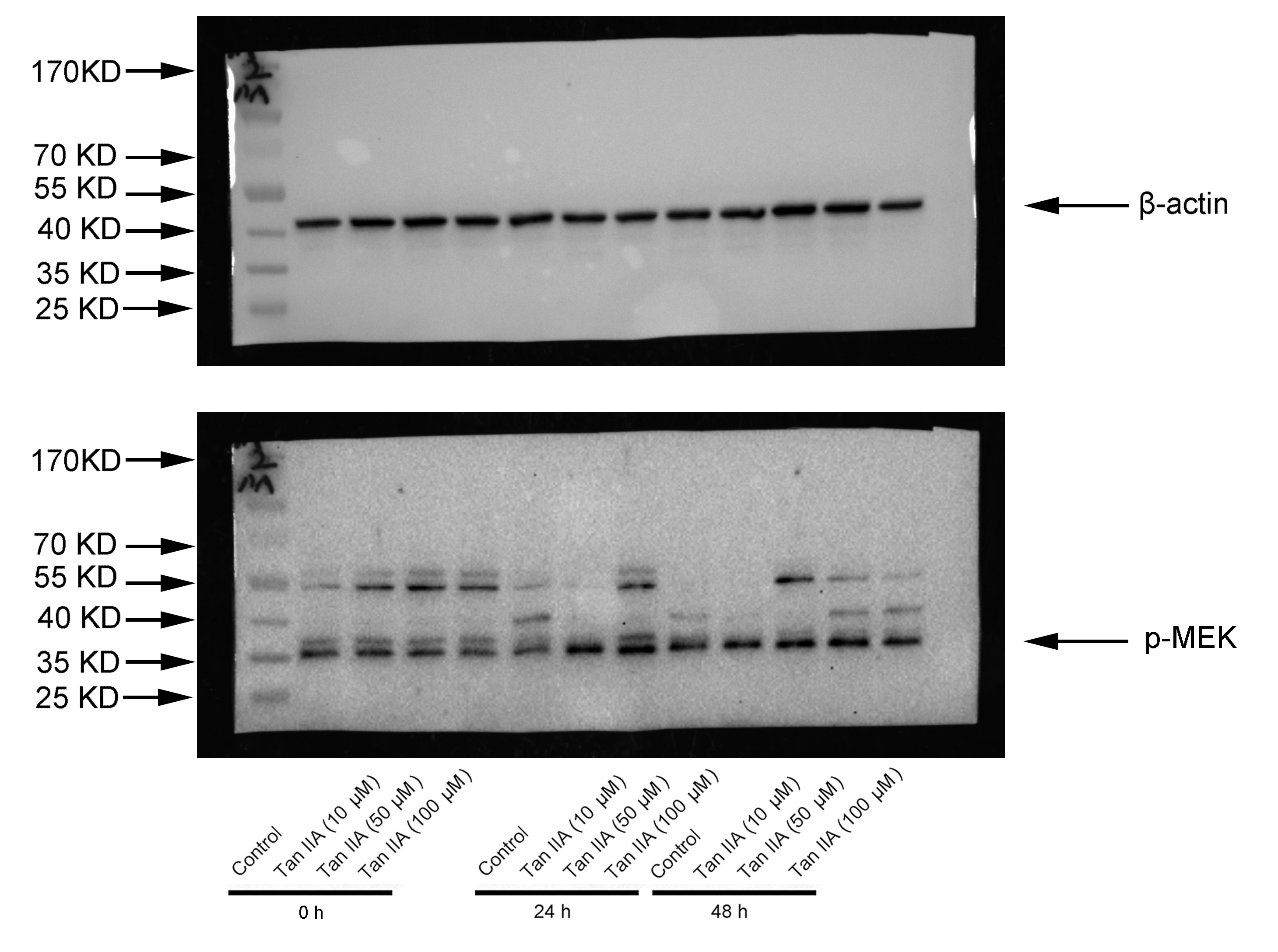
**

Uncropped western blots of Fig 5B.

**
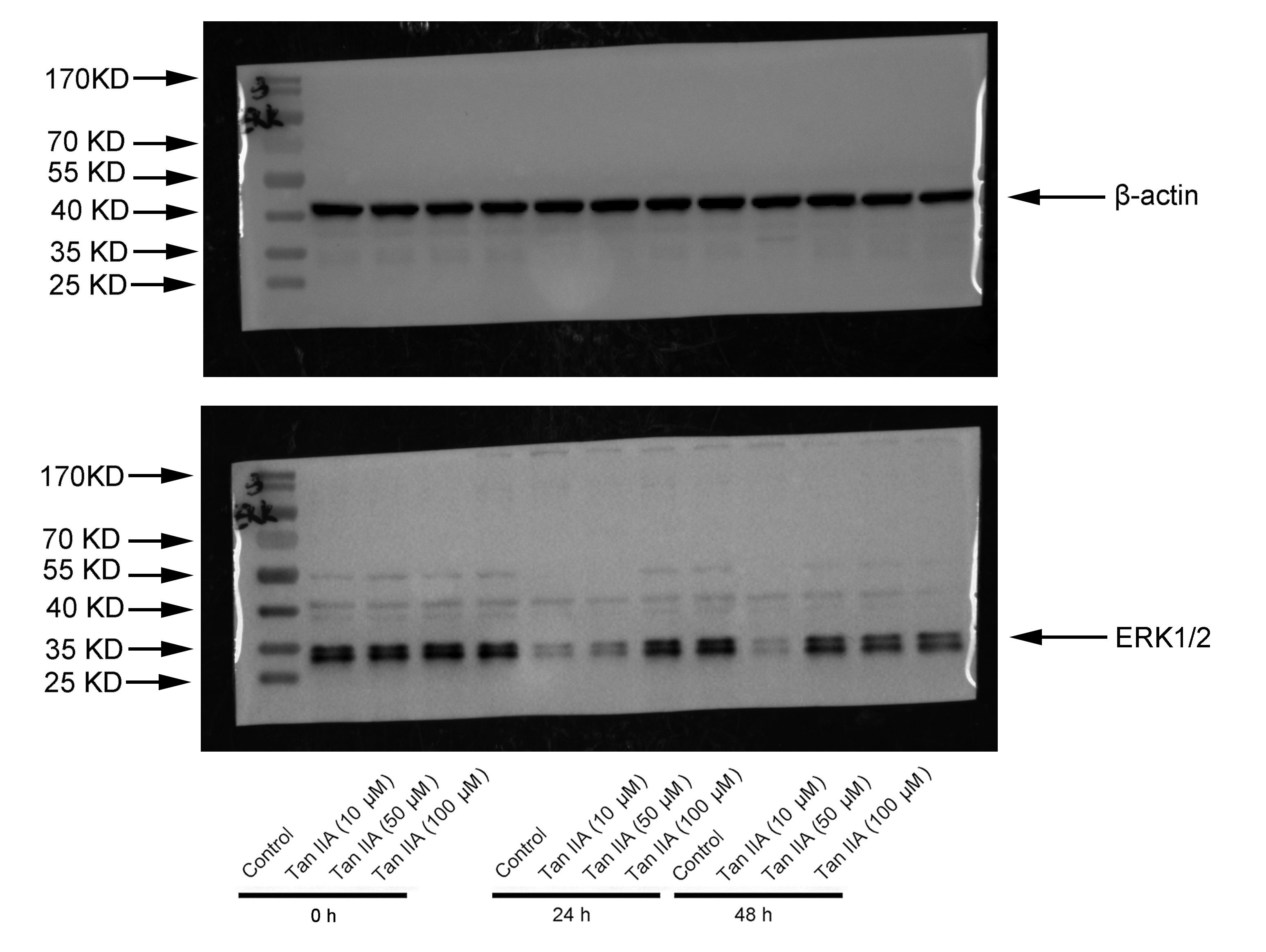
**

Uncropped western blots of Fig 5C.

**
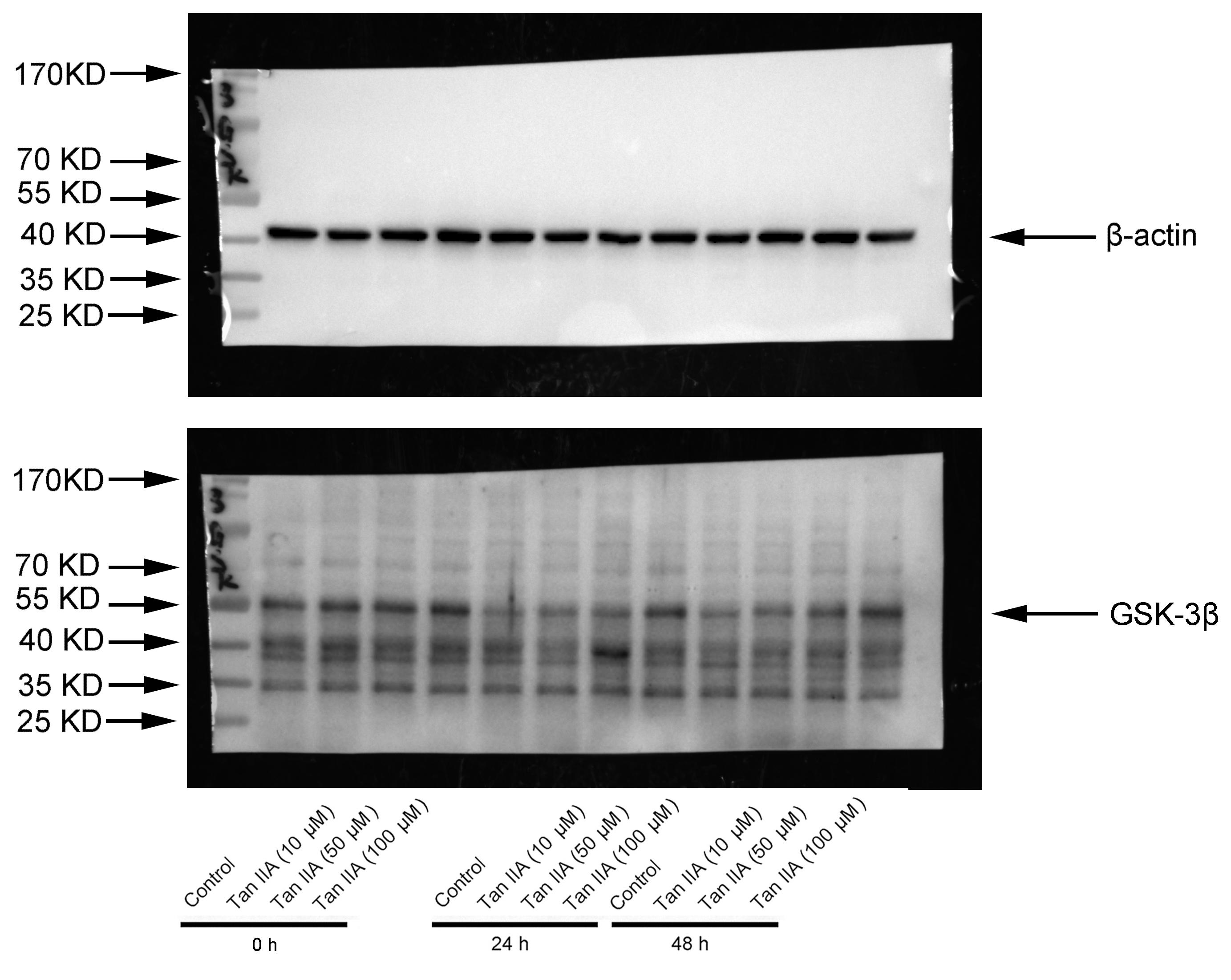
**

Uncropped western blots of Fig 6E.

**
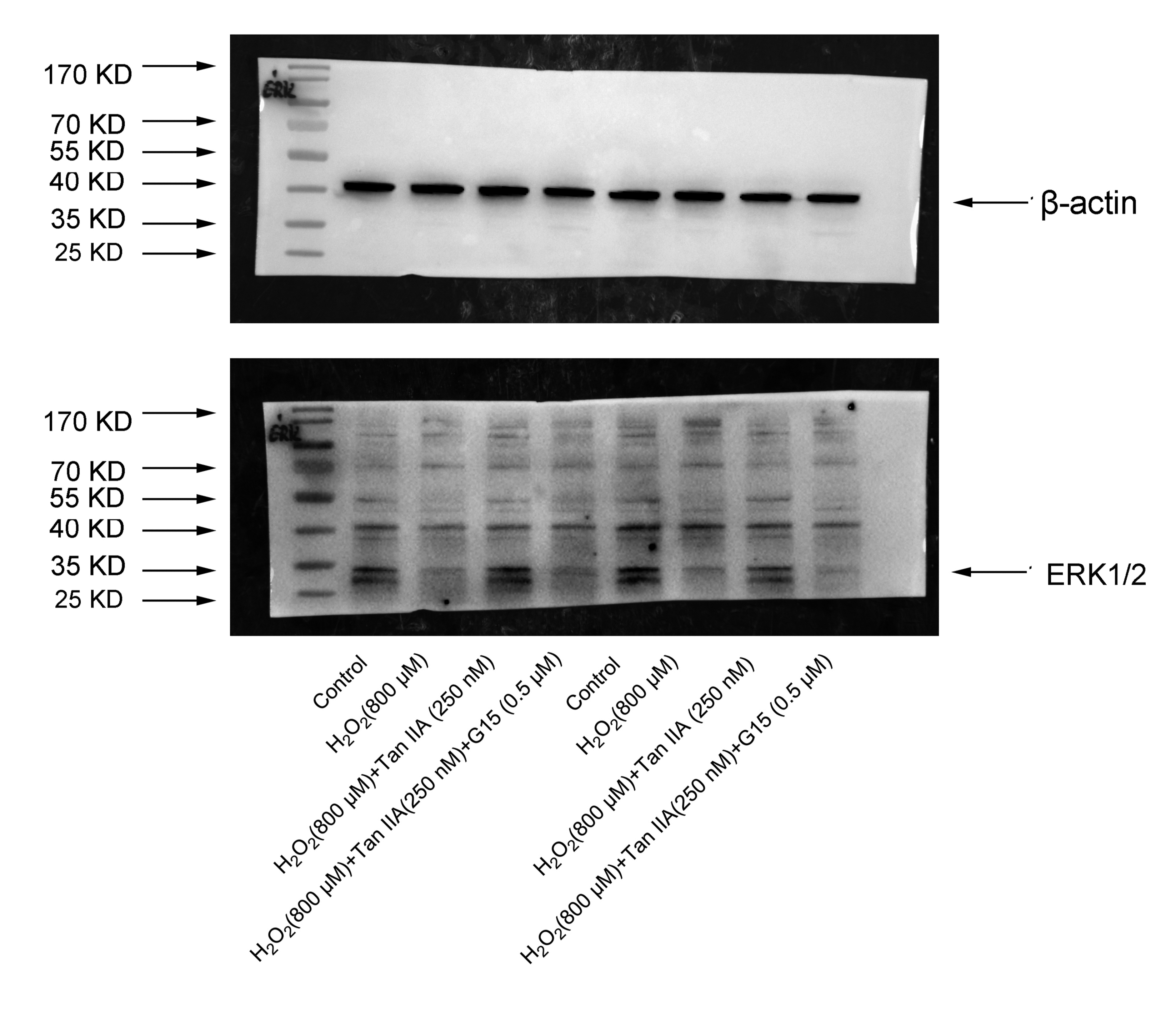

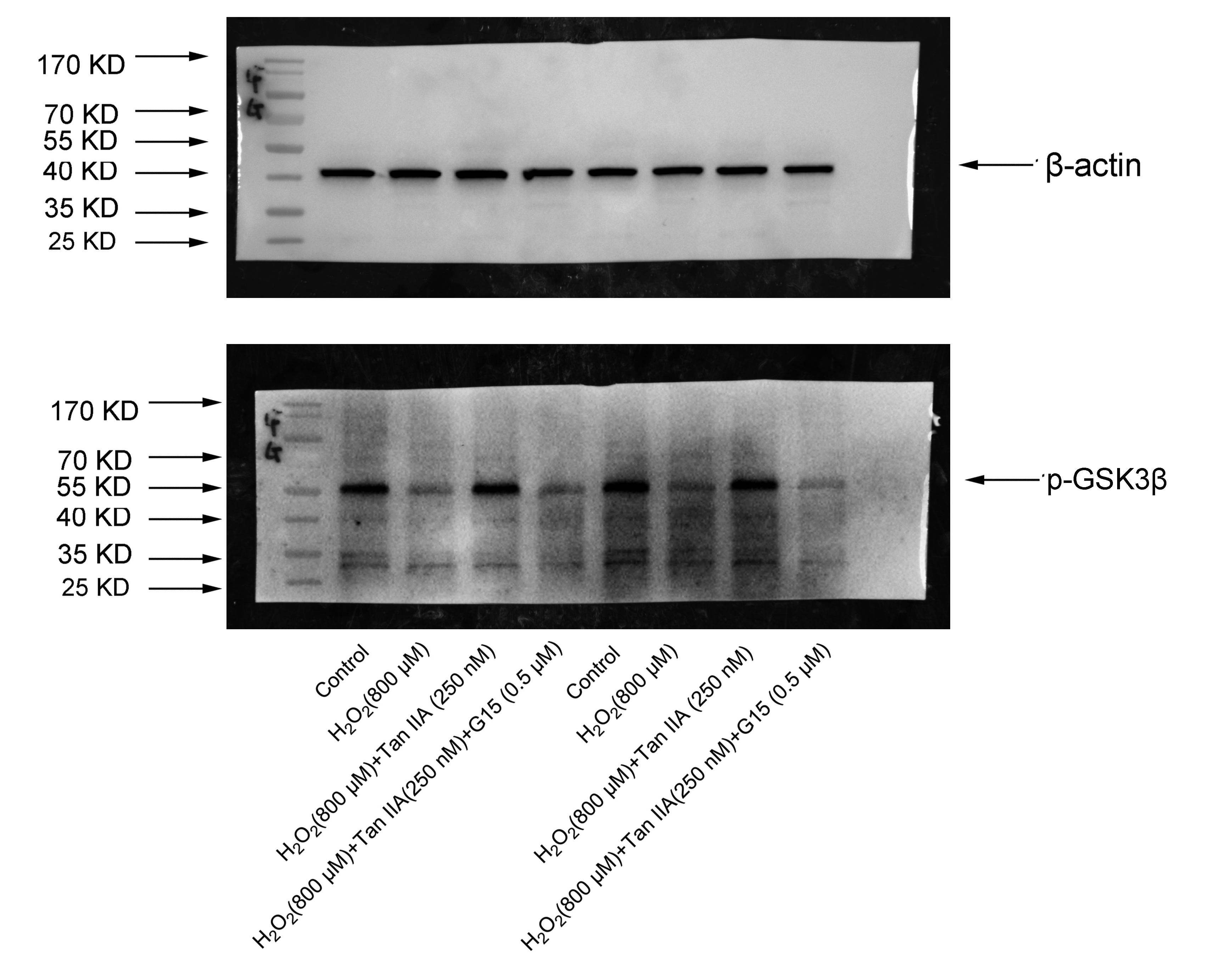

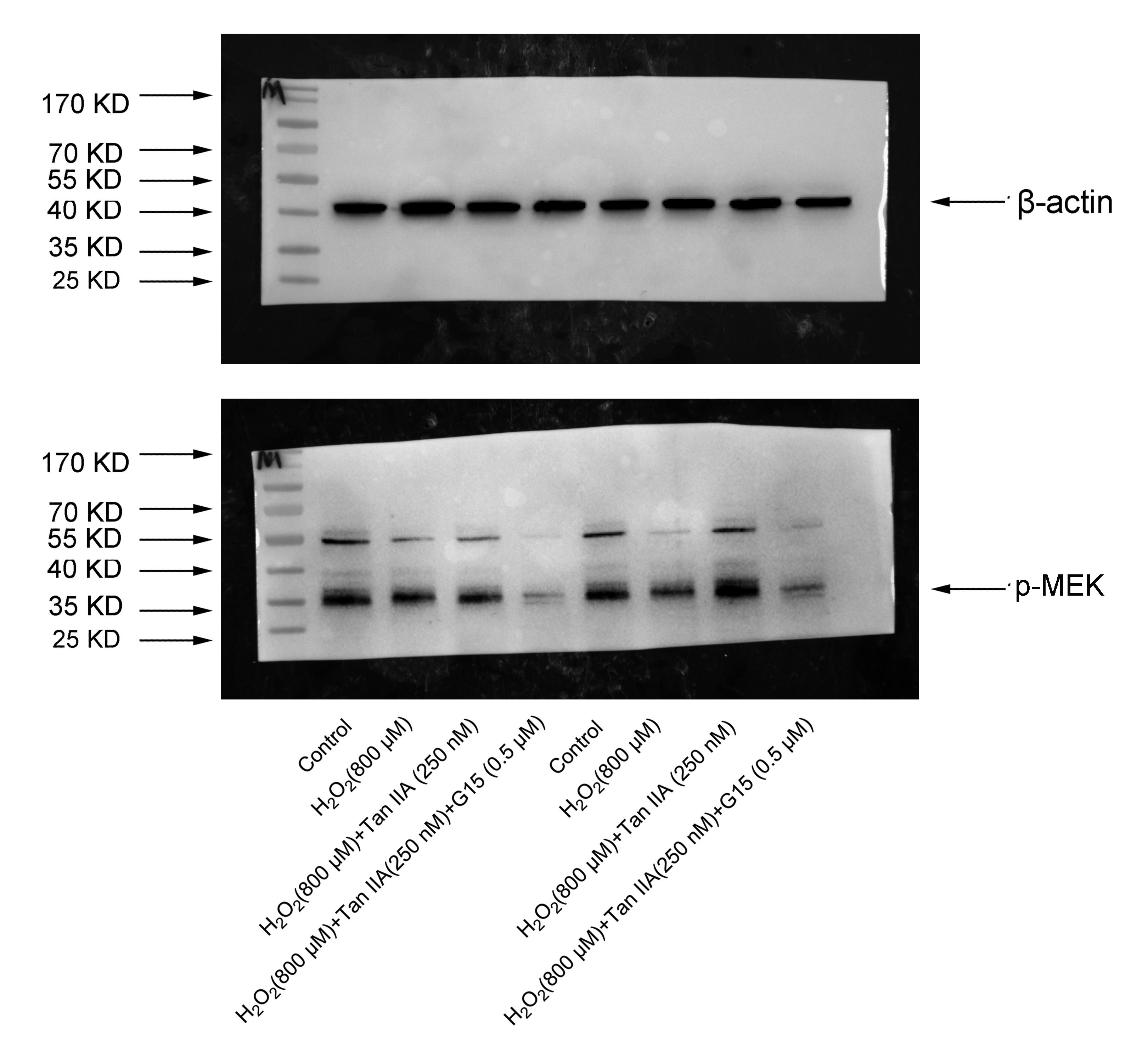
**

**
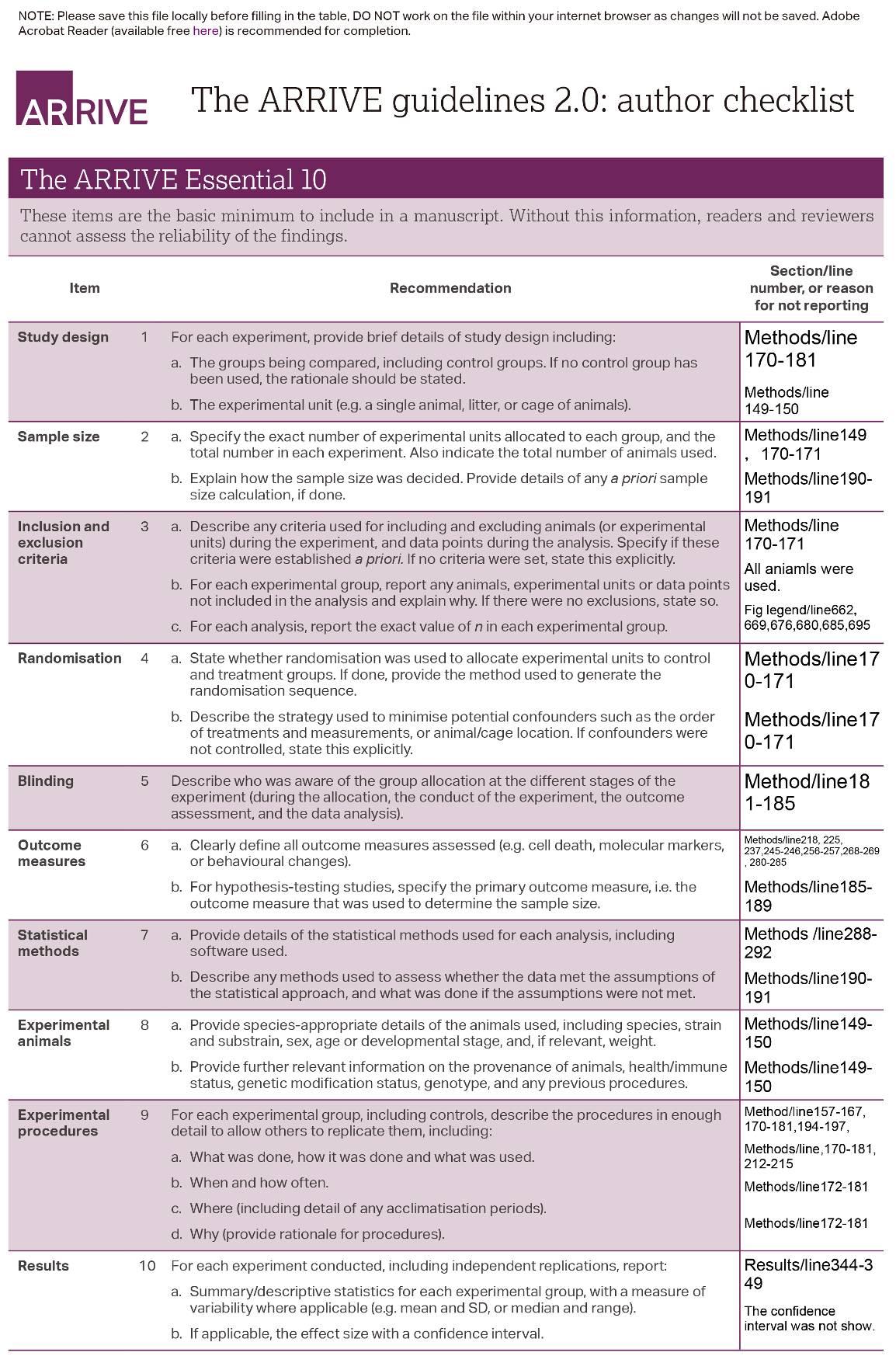
**

**
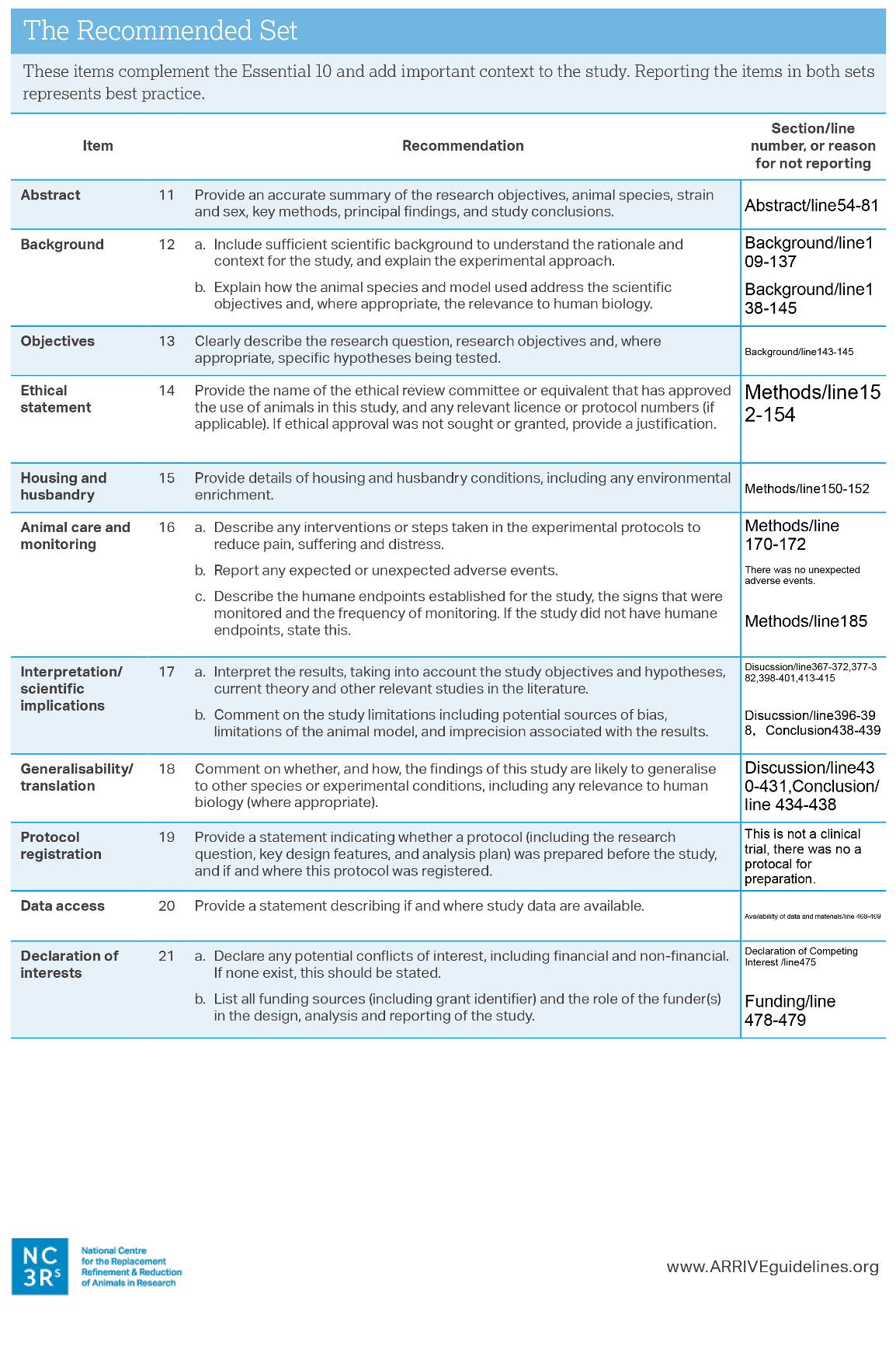
**
